# Potential vector species of carp edema virus (CEV)

**DOI:** 10.1111/jfd.13000

**Published:** 2019-04-23

**Authors:** Marek Matras, Magdalena Stachnik, Ewa Borzym, Joanna Maj‐Paluch, Michal Reichert

**Affiliations:** ^1^ Department of Fish Diseases National Veterinary Research Institute Pulawy Poland

**Keywords:** carp oedema virus, common carp, koi sleepy disease, vectors

## Abstract

During a PCR‐based CEV survey in Poland in 2015–2017, the virus was detected in many farms both in clinical and asymptomatic cases and in common as well as in koi carp (*Cyprinus carpio*). In order to evaluate the potential carrier role of fish species that share the same habitats with carp, an experimental trial was performed. Investigations carried out on specimens of bleak (*Alburnus alburnus*), crucian carp (*Carassius carassius*), European perch (*Perca fluviatilis*), Prussian carp (*Carassius gibelio*), roach (*Rutilus rutilus*) and tench (*Tinca tinca*) cohabited with CEV‐infected carp yielded positive results. These species of fish were experimentally cohabited with CEV‐infected common carp at a temperature of 16°C ± 1. Material from the brain, gills, spleen, kidneys, intestine and skin was investigated for the presence of CEV DNA. Similar investigations were performed with uninfected fish designated controls. Samples were tested for CEV by qPCR.

## 1 INTRODUCTION

Carp oedema virus (CEV) was first detected in Japan in the 1970s, where it caused outbreaks of disease accompanied by high mortality among juvenile *Cyprinus carpio* of the koi variety (Murakami, Shitanaka, Toshida, & Matsuzato, 1976). In recent years, CEV has been isolated in Japan from koi manifesting sleepy behaviour and the disease has been termed koi sleepy disease (KSD). In Europe, in the years subsequent to its definition in Japan, KSD cases in common carp have been noted as well as in koi (Way & Stone, 2013). During a PCR‐based CEV survey in Poland in 2015–2017, the infection was detected in many farms in clinical and asymptomatic cases in both carp varieties (Matras et al., 2017). Although the virus has been present in aquaculture for many years, there is still a lack of knowledge concerning factors of virus transmission. Its vectors are yet to be elucidated; for example, other fish species kept together with common carp. Understanding of the pathways that have led to the transmission of the virus through the whole *Cyprinus carpio* species is an important element in the prevention of the spread of CEV/KDS. The mode of horizontal transmission of fish viruses may be direct (fish to fish) or by vectors, water being the major abiotic vector. However, hypothetically, animate vectors, for example other fish species, parasitic invertebrates and piscivorous birds and mammals, can also be involved in transmission. There is a lack of publications in the available literature on the potential role of other fish species commonly kept with carp in ponds as vectors of CEV infection. The role of different fish species as vectors of virus transmission is well known in the cases of viral haemorrhagic septicaemia virus (VHSV) and infectious haematopoietic necrosis virus (IHNV) (European Food Safety Authority (EFSA) 2008; Olesen & Vestergård Jørgensen, 1982; Peters & Neukirch, 1986; Bootland & Leong, 1999), and this knowledge is gradually being expanded in koi herpesvirus (KHV) infection (Bergmann et al., 2009; Kempter et al., 2009; Minamoto et al., 2010), but there is no such knowledge about the role of animate vectors of CEV infection. A better understanding of the mechanisms of transmission of koi sleepy disease is important and the knowledge of animate vectors will be helpful in measures aiming to limit the spread of the virus.

## 2 MATERIALS AND METHODS

### 2.1 Experimental trials

Fish from six different species were experimentally cohabited with CEV‐infected common carp (*Cyprinus carpio*) in two separate trials. In the first cohabitation experiment, 30 specimens each of bleak (*Alburnus alburnus*), crucian carp (*Carassius carassius*), Prussian carp (*Carassius gibelio*), roach (*Rutilus rutilus*) and tench (*Tinca tinca*) were introduced to tanks with 20 CEV‐infected carp. Control fish (30 in each species) were cohabited with naïve carp. Both groups were transferred after 12 hr of cohabitation with CEV‐infected and naïve fish to separate 600‐L tanks with filtered and aerated water at a temperature of 16 ± 1°C. At 3, 7, 14 and 21 days post‐exposure, two fish per species were killed by immersion into a 0.5 g/L tricaine solution (Sigma‐Aldrich) and samples of brain, gills, spleen, kidney, intestine and skin were collected. At the same time intervals, samples from control group fish were collected. In the second experimental trial, bleak, crucian carp, European perch (Perca fluviatilis), Prussian carp and roach were cohabited with CEV‐infected carp for 72 hr. At 7‐day intervals (at 7, 14, 21, 28, 35 and 42 days post‐exposure), three fish per species were killed and samples were taken as described above. At the same time intervals, samples from control group fish were collected.

### 2.2 DNA extraction

The tissue samples were homogenized and total DNA was extracted using a QIAamp DNA Mini Kit (Qiagen, Germany) according to the manufacturer instructions. The DNA was eluted in 100 µl of acetate ethylenediaminetetraacetic acid (AE) buffer and stored at − 80°C before testing.

### 2.3 Molecular identification

All samples were initially tested by real‐time PCR using a QuantiTect Probe commercial PCR kit and a Rotor‐Gene Q thermal cycler (Qiagen, Germany). The protocol adopted was developed originally by the Centre for Environment, Fisheries and Aquaculture Science (CEFAS) in Weymouth, UK. Assays were performed in a 20 µl reaction volume consisting of 500 nM forward CEV qFor1 (5′‐AGTTTTGTAKATTGTAGCATTTCC‐′3) and reverse CEV qRev1 (5′‐GATTCCTC AAGGAGTTDCAGTAAA‐′3) primers, 200nM CEV qProbe1 (5′‐AGAGT TTGTTTCTTGCCATACAAACT‐′3), 1 × PCR buffer mix and 5 µl of the DNA extracted above. Assays were performed using the CFX Connect Real‐Time PCR Detection System (Bio‐Rad, USA). The samples were initially held at 50°C for 2 min followed by 10 min at 95°C and were then put through 50 temperature cycles of 15 s at 95°C and then held for 1 min at 55°C. The CEFAS primers and the above real‐time PCR method have been successfully used in infected tissue samples from koi and common carp (Matras et al., 2017). CVI NL‐13013096 and CVI NL‐14009726 genetic materials (courtesy of Dr Olga Haenen from the NRL for Fish, Crustacean and Shellfish Diseases in the Netherlands) were used as a positive control in the molecular identification.

## 3 RESULTS

The presence of carp oedema virus nucleic acid after 12 hr of cohabitation was detected by the qPCR method on the 7th‐day post‐experimental exposure in tench and crucian carp, in both cases in the gills. On the 14th day post‐exposure, the presence of CEV nucleic acid was found the gills of in two tench and one Prussian carp. We observed the presence of virus on the 21th day post‐cohabitation in two Prussian carp, also only in samples from the gills (Table 1). In the second experimental challenge with 72 hr of cohabitation, we observed the presence of CEV DNA in 7 out of 15 killed fish (one crucian carp, one Prussian carp, one European perch, two roach and two bleak) on the 7th day post‐exposure. In samples from this period of infection, we obtained positive results in gills and in the skin (Table 2). On day 14 after the transfer of the fish to separate tanks, the presence of CEV was confirmed in nine fish, the sample tissue being the gills of two Prussian carp, one roach, one bleak and one European perch and also in the skin of one crucian carp and one European perch. In one crucian carp, we detected virus nucleic acids in both samples: from gills and skin. On the 21st day post‐exposure, only two samples were positive: one from the gills of a crucian carp and the second from the skin of a roach. CEV DNA was detected 28 days post‐infection in the gills of three fish (two crucian carp and one European perch) and in the skin of four fish (one Prussian carp, one roach and two bleaks). In samples originating from the next time interval, we obtained positive results in 12 fish. We observed the presence of virus DNA in the gills and skin of roach, but we also had positive samples from the skin in 11 fish (Table 2). In samples collected 42 days post‐exposure, we confirmed CEV DNA in five fish. In samples originating from control fish, we did not detect the presence of carp oedema virus. In samples from bleak and roach, in trials with 12‐hr exposure, we did not detect carp oedema virus. But in the experimental challenge with 72 hr cohabitation, we confirmed virus DNA in samples collected from all species used in the experiment. We did not observe any clinical signs or mortality in fish after cohabitation with CEV‐infected common carp to the end of the experiment.

**Table 1:** Real‐time PCR‐positive results (protocol developed originally in CEFAS‐Weymouth, UK) in fish cohabitated 12 hr with CEV‐infected common carp at 16ºC

Species	Tissue	Results of real‐time PCR/*C_t_*	Days post‐exposure
Tench	Brain, kidney, spleen, intestine, skin	–	7
	Gill	+/39.11	
Crucian carp	Brain, kidney, spleen, intestine, skin	–	
	Gill	+/39.74	
Tench	Brain, kidney, spleen, intestine, skin	–	14
	Gill	+/34.06	
Tench	Brain, kidney, spleen, intestine, skin	–	
	Gill	+/43.83	
Prussian carp	Brain, kidney, spleen, intestine, skin	–	
	Gill	+/38.78	
Prussian carp	Brain, kidney, spleen, intestine, skin	–	21
	Gill	+/41.44	
Prussian carp	Brain, kidney, spleen, intestine, skin	–	
	Gill	+/37.16	

**Table 2:** Real‐time PCR‐positive results (protocol developed originally in CEFAS‐Weymouth, UK) in fish cohabitated 72 hr with CEV‐infected common carp at 16ºC

Species	Tissue	Results of real‐time PCR/*C_t_*	Days post‐exposure
Crucian carp	Brain, gill, kidney, spleen, intestine	–	7
	Skin	+/42.22	
Prussian carp	Brain, gill, kidney, spleen, intestine	–	
	Skin	+/43.19	
Roach	Brain, kidney, spleen, intestine, skin	–	
	Gill	+/42.58	
	Skin	+/42.16	
Roach	Brain, kidney, spleen, intestine, skin	–	
	Gill	+/38.84	
Bleak	Brain, kidney, spleen, intestine, skin	–	
	Gill	+/42.24	
Bleak	Brain, kidney, spleen, intestine	–	
	Gill	+/42.71	
	Skin	+/42.61	
European perch	Brain, kidney, spleen, intestine, skin	–	
	Gill	+/41.75	
Crucian carp	Brain, kidney, spleen, intestine	–	14
	Gill	+/42.64	
	Skin	+/42.66	
Crucian carp	Brain, kidney, spleen, intestine, skin	–	
	Gill	+/41.69	
Prussian carp	Brain, kidney, spleen, intestine, skin	–	
	Gill	+/41/42	
Prussian carp	Brain, gill, kidney, spleen, intestine	–	
	Skin	+/42.75	
Prussian carp	Brain, kidney, spleen, intestine, skin	–	
	Gill	+/42.21	
Roach	Brain, kidney, spleen, intestine, skin	–	
	Gill	+/40.24	
Bleack	Brain, kidney, spleen, intestine, skin	–	
	Gill	+/40.61	
European perch	Brain, kidney, spleen, intestine, skin	–	
	Gill	+/41.24	
European perch	Brain, gill, kidney, spleen, intestine	–	
	Skin	+/42.60	
Crucian carp	Brain, kidney, spleen, intestine, skin	–	21
	Gill	+/41.49	
Roach	Brain, gill, kidney, spleen, intestine	–	
	Skin	+/41.66	
Crucian carp	Brain, gill, kidney, spleen, intestine	–	28
	Gill	+/42.53	
Crucian carp	Brain, gill, kidney, spleen, intestine	–	
	Gill	+/40.34	
Prussian carp	Brain, gill, kidney, spleen, intestine	–	
	Skin	+/40.83	
Roach	Brain, gill, kidney, spleen, intestine	–	
	Skin	+/37.67	
Bleak	Brain, gill, kidney, spleen, intestine	–	
	Skin	+/38.18	
Bleak	Brain, gill, kidney, spleen, intestine	–	
	Skin	+/38.79	
European perch	Brain, kidney, spleen, intestine, skin	–	
	Gill	+/40.08	
Crucian carp	Brain, gill, kidney, spleen, intestine	–	35
	Skin	+/38.22	
Prussian carp	Brain, gill, kidney, spleen, intestine	–	
	Skin	+/39.14	
Prussian carp	Brain, gill, kidney, spleen, intestine	–	
	Skin	+/38.51	
Roach	Brain, kidney, spleen, intestine	–	
	Gill	+/38.66	
	Skin	+/39.26	
Roach	Brain, gill, kidney, spleen, intestine	–	
	Skin	+/39.02	
Roach	Brain, gill, kidney, spleen, intestine	–	
	Skin	+/35.32	
Bleak	Brain, gill, kidney, spleen, intestine	–	
	Skin	+/36.35	
Bleak	Brain, gill, kidney, spleen, intestine	–	
	Skin	+/37.93	
Bleak	Brain, gill, kidney, spleen, intestine	–	
	Skin	+/36.49	
European perch	Brain, gill, kidney, spleen, intestine	–	
	Skin	+/39.15	
European perch	Brain, gill, kidney, spleen, intestine	–	
	Skin	+/39.15	
European perch	Brain, gill, kidney, spleen, intestine	–	
	Skin	+/38.38	
Crucian carp	Brain, gill, kidney, spleen, intestine	–	42
	Skin	+/39.14	
Crucian carp	Brain, gill, kidney, spleen, intestine	–	
	Skin	+/40/15	
Prussian carp	Brain, gill, kidney, spleen, intestine	–	
	Skin	+/41.36	
Prussian carp	Brain, kidney, spleen, intestine	–	
	Gill	+/42.46	
	Skin	+/40.38	
Bleak	Brain, kidney, spleen, intestine, skin	–	
	Gill	+/41.51	

## 4 DISCUSSION

The results of our investigation confirmed that CEV‐infected common carp are able to transmit the virus to other fish species. In the literature, there are published results indicating that KSD‐afflicted common carp, as well as the ornamental koi variety, are able to transmit the viral disease to other fish from the *Cyprinus carpio* species (Adamek et al., 2017). However, we showed that during cohabitation an infective agent is transmitted from CEV‐infected common carp to heterospecific fish of the bleak, crucian carp, European perch, Prussian carp, roach and tench species. In fish from six species used in our study, we observed neither clinical signs nor mortality. In the first experimental trial, we observed the presence of CEV nucleic acids in samples 7 days post‐exposure. Interestingly, 3 days post‐exposure all collected samples were negative. Carp oedema virus was confirmed only in the gills of 17.5% of fish sampled after experimental challenge. It seems that 12 hr of cohabitation, although a short period of time, is enough for transmission of CEV from infected common carp to vector species. Our data, concerning the time and possibility of virus transmission, are in line with the results of other authors, where 6 hr of cohabitation was enough to transmit the CEV infection to other, naïve koi and specific pathogen free (SPF) common carp (Haenen et al., 2016). In an experimental trial with 72 hr of cohabitation, the presence of CEV DNA was observed at 7, 14, 21, 28, 35 and 42 days post‐exposure in samples of gills and skin. It seems that a longer time of cohabitation of naïve fish from different species with CEV‐infected common carp causes transmission of the virus to different organs. Probably, that virus DNA was first detected in gills and then in skin is explained by the relative sizes of fish skin surface area and gill area. For example, common carp of about 20 g weight have 9,220‐mm^2^ gill area and 5,753‐mm^2^ skin surface area (Ling et al., 2008; Oikawa & Itazawa, 1985). The constant flow of water and size of the gill area are the explanation for why after 12 hr of cohabitation CEV DNA was found only in gill samples. In experimental virus transmission studies on CEV‐infected common carp, Adamek et al. (2017) confirmed that gill tissue had higher virus loads than other organs such as kidney, spleen, skin and gut. In our study, we focused on the distribution of CEV DNA in organs and tissues of potential vector fish that had contact with CEV‐infected common carp. We found the presence of virus DNA in skin and gills, whereas all samples from brain, kidney, spleen and intestine were negative. We noted the lowest *C_t_* values (*C_t_* = 35.32) in a sample of roach skin.

This study provided data on the distribution of carp oedema virus in organs and tissues of potential vector species, but there is still a need for further investigation concerning transfer of the pathogen from vectors to naïve fish and other fish species that are kept together with common carp as additional fish. There are two main groups of other species reared in polyculture with carp. The first group consists of predatory species such as pike (*Esox Lucius*), wels catfish (*Silurus glanis*), pike perch (*Sander lucioperca*) and European perch, and the second group includes cyprinid species such as crucian carp, tench, grass carp (*Ctenopharyngodon idella*), silver carp (*Hypophthalmichthys molitrix*), bighead carp (*Hypophthalmichthys nobilis*), ide (*Leuciscus idus*), asp (*Leuciscus aspius*), chub (*Squalius cephalus*), vimba bream (*Vimba vimba*), barbel (*Barbus barbus*) and common nase (*Chondrostoma nasus*). Pathways that have led to the transmission of the CEV throughout fish farms are also similar like in the course of infection with koi herpes virus (CyHV‐3). There is evidence to that other fish species are potential vectors of KHV. Bergmann et al. (2009) has been detected KHV by nested PCR in several different varieties of goldfish as well as grass carp (*Ctenopharyngodon idella*), ide (*Leuciscus idus*) and ornamental catfish (*Ancistrus*sp.). KHV was detected by PCR in Russian sturgeon (*Acipenser gueldenstaedtii*) and Atlantic sturgeon (*A. oxyrinchus*) from fish farms in Northern Poland (Kempter et al., 2009). Bergmann et al. (2010) reported the replication of KHV in goldfish after experimental infection by immersion. Lack of clinical signs and mortality, and the presence of CEV only in external tissues in our study suggested that fish other than common carp are vectors which do not actively replicate the virus. In our experimental study, we used six species but we can suspect that potential vector species in the transmission of CEV are all other fish that are reared in polyculture with common carp. According to Way et al. (2017), animal vectors and carp acting as carriers of the virus will potentially play a major role in the spread of CEV to naïve populations. This preliminary study confirms this thesis that bleak, crucian carp, European perch, Prussian carp, roach and tench may play a role as animal vectors of CEV infection. In species which were cohabited with CEV‐infected carp for 72 hr, we detected viral DNA until the 42nd day. These data confirmed that even a short contact time of vector species with infected fish may potentially be one way of virus transmission with a long period until elimination from the vectors.

This knowledge concerning the mechanism of spread of the CEV will be useful for veterinary services and breeders to limit the spread of CEV to naïve populations, making future measures for the eradication of the disease more reliable.

## CONFLICT OF INTEREST

The authors declare no conflict of interest.
